# From lactate to lactylation: novel pathological mechanisms and potential therapeutic targets for high-altitude cerebral oedema

**DOI:** 10.1017/erm.2026.10046

**Published:** 2026-04-06

**Authors:** Chaoyi Duan, Siyu Li, Likun Yao, Xinjie Zhang, Yansheng Ding, BiWen Peng

**Affiliations:** 1 Wuhan University, China; 2 Weifang Maternal and Child Health Hospital, China

**Keywords:** high-altitude cerebral oedema, lactate, lactylation, neuroinflammation

## Abstract

**Background:**

High altitude cerebral edema (HACE), a fatal terminal stage of acute mountain sickness (AMS), is triggered by rapid exposure to hypoxia at high altitudes. The pathophysiology of HACE is complex, involving multiple key processes including energy metabolism disorders, oxidative stress, blood-brain barrier (BBB) injury, and neuroinflammation, all of which interact to drive disease progression. Lactylation, a novel epigenetic regulatory mechanism discovered in 2019, provides a fresh perspective for HACE research.

**Methods:**

This study integrates the latest research findings on the pathophysiology of HACE, lactate metabolism, and the role of lactylation in hypoxia-related diseases (such as cancer and ischemic-hypoxic diseases). It focuses on analyzing the potential molecular mechanisms of lactylation in HACE, including its regulation of the HIF-1α/NF-κB axis, inflammation, and metabolism, and discusses existing lactylation regulation strategies.

**Results:**

In HACE, hypoxia-driven glycolysis elevates lactate, promoting protein lactylation (e.g., NuRD complex in microglia, which is correlated with proinflammatory cytokines). Lactylation may regulate HIF-1α/NF-κB axis, inflammation, and metabolism in HACE pathogenesis. Currently, methods such as the inhibition of lactate dehydrogenase (LDH) /monocarboxylate transporters and the use of histone deacetylase inhibitors have been proven effective in regulating lactylation.

**Conclusion:**

Lactylation is a key link connecting metabolic disorders and neuroinflammation in HACE. However, the dual role of lactate in neuroprotection and neuroinjury under hypoxic conditions still requires further exploration. Future research should focus on deciphering the molecular networks related to HACE and developing precise intervention strategies to provide new directions for HACE treatment.

## Introduction

Currently, acute mountain sickness (AMS) remains a significant safety problem both for the tourism industry and for individuals entering high altitude. The hypobaric hypoxia has been demonstrated to elevate the risk of suffering from AMS (Ref. 1). AMS is characterised by a range of symptoms, including but not limited to headache, loss of appetite and fatigue. In severe cases, this condition can develop without warning into a form of cerebral oedema known as high-altitude cerebral oedema (HACE), which can ultimately prove fatal. HACE is a serious and life-threatening condition caused by rapid elevation to high altitude, with a prevalence of 0.5%–1% between 4200 m and 5500 m, increasing to 30%–50% between 5500 m and 8000 m, and has a high mortality rate (Ref. 2). It depends on altitude, the rate of altitude climb, the duration of exposure, physical status, mood and individual susceptibility (Refs 2, 3, 4, 5). The diagnosis can be made on the basis of clinical findings, brain computed tomography (CT) and magnetic resonance imaging (MRI). In patients with HACE, MRI revealed significant cerebral oedema with microvascular haemorrhages in the corpus callosum and white matter regions, with evidence of both cytotoxic and vasogenic oedema (Ref. 6). However, in reality, HACE progresses rapidly, is not easily detected, and usually progresses to severe HACE by the time medical attention is sought.

When an organism is under hypoxia, anaerobic glycolysis in the cytoplasm is the main source of cellular ATP, and lactate may play an important role in the development of HACE disease. In 2019, Zhao’s team discovered a new epigenetic modification, namely, lactylation, whereby lactate can serve as a substrate for modifying histone lysine residues and regulating downstream gene expression (Ref. 7). Lactylation is involved in the development of many diseases, such as cancer, ischaemic–hypoxic diseases and metabolic diseases, which are closely related to the hypoxic state of the organism (Refs 8, 9, 10). At present, although there are few studies related to lactylation in HACE disease, one study reported that sustained 6000 m hypoxic exposure for 12 h increased lactate concentration and lactylation levels in HACE mice model, and lactylation appeared preferentially in protein complexes (Ref. 11).

This review takes the pathophysiological mechanisms of HACE as a starting point for identifying the role of lactate and lactylation in the hypoxic state of the organism to demonstrate the importance of lactylation in HACE and to identify potential targets for the treatment and prevention of the disease.

## Pathophysiological mechanism of HACE

**Figure 1.**
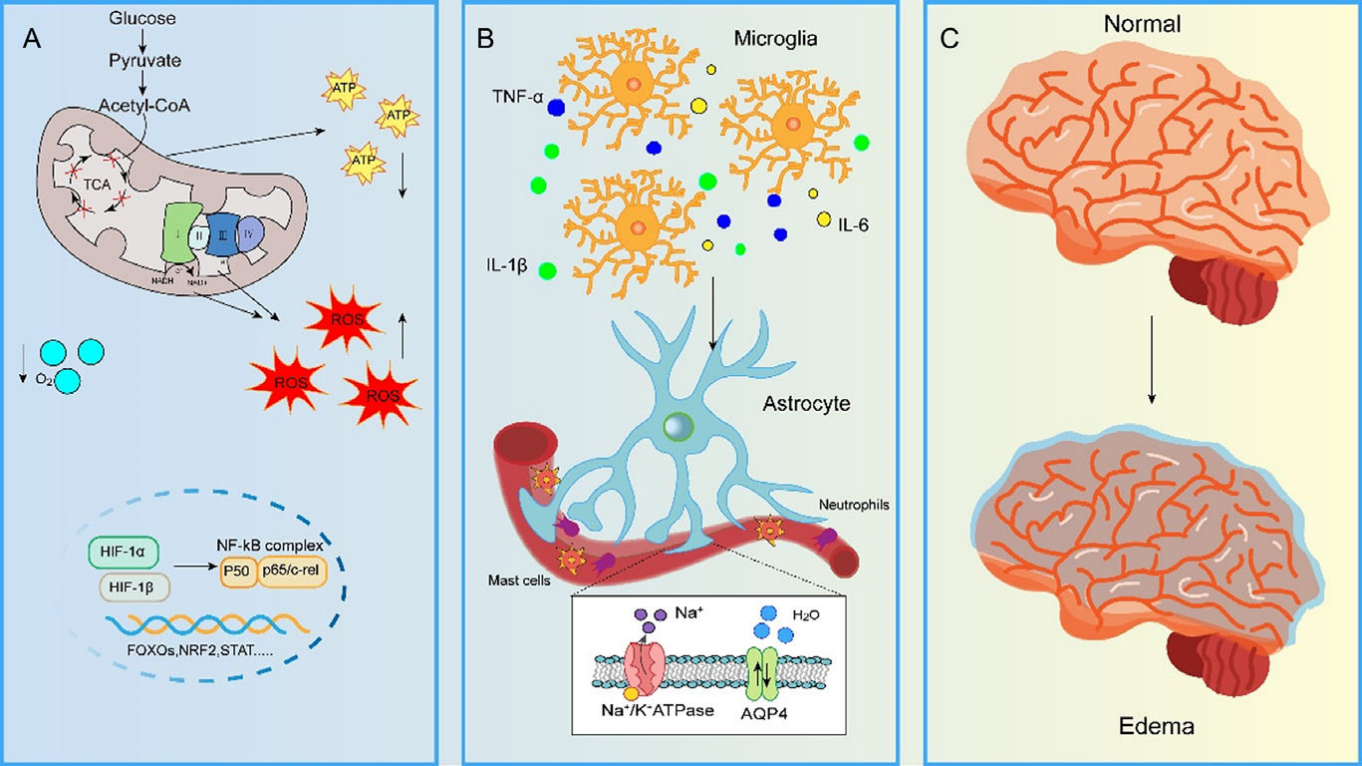
Pathophysiological mechanisms underlying high-altitude cerebral oedema (HACE).(A) Under hypoxic conditions, mitochondrial oxidative phosphorylation is impaired, leading to reactive oxygen species (ROS) accumulation and ATP depletion. Stabilisation of hypoxia-inducible factor-1α (HIF-1α) activates the nuclear factor-κB (NF-κB) signalling pathway, thereby promoting transcription of pro-inflammatory and stress-response genes (FOXO3, NRF2, STAT). (B) Activated microglia release pro-inflammatory cytokines (TNF-α, IL-1β, IL-6), initiating a neuroinflammatory cascade that recruits and activates astrocytes. Concurrently, peripheral immune cells – including neutrophils and mast cells – migrate into the central nervous system (CNS). The inset depicts astrocyte-mediated ion dysregulation resulting from dysfunction of Na^+^/K^+^-ATPase and aquaporin-4 (AQP4), contributing to cytotoxic oedema. (C) Schematic illustration of brain morphological changes progressing from normal anatomy to cerebral oedema, reflecting the integrated consequences of metabolic disruption, neuroinflammation and blood–brain barrier compromise.

During acute exposure to hypoxia, the body maintains an oxygen supply–demand balance through compensatory cardiopulmonary enhancement (e.g., increased respiratory rate and elevated cardiac output). However, when the duration of hypoxia exceeds the physiological regulatory threshold, aberrant fluctuations in cerebral blood flow coupled with disruption of vascular endothelial integrity collectively lead to cerebral oedema and intracranial hypertension. This pathological progression involves multiple molecular mechanisms. As shown in Figure 1, disturbances in energy metabolism, oxidative stress, blood–brain barrier (BBB) injury and neuroinflammation are involved in the pathogenesis of HACE. Nevertheless, the underlying mechanisms remain incompletely understood.

### HACE and energy metabolism disorder

The pathological mechanism of HACE is closely associated with energy metabolism disorder, with a dual pathological process of mitochondrial dysfunction and compensatory imbalance in glucose metabolism (Refs 12, 13). Under hypoxic stress, the mitochondrial oxidative phosphorylation system is significantly suppressed, directly leading to a decrease in ATP production (Figure 1). Notably, dysfunction of the electron transport chain not only exacerbates the energy crisis but also triggers substantial generation of reactive oxygen species (ROS). Experimental evidence indicates that the mitochondrial membrane potential is markedly decreased, with ultrastructural observations revealing reduced crista density and mitochondrial vacuolisation, which directly impair fundamental neuronal activity (Ref. 14). On the other hand, cells compensate by enhancing the glycolytic metabolic pathway, manifested by upregulated activities of lactate dehydrogenase (LDH) and pyruvate kinase, alongside a concomitant surge in intracellular lactate levels and elevated ATPase activity (Ref. 15). This metabolic reprogramming exacerbates central nervous system damage through two synergistic pathways: Firstly, insufficient ATP supply leads to dysfunction of the Na^+^/K^+^-ATPase pump, which causes intracellular Na^+^ accumulation and K^+^ efflux, leading to astrocytic swelling (via elevated osmolarity and water influx), impaired endothelial tight junctions, increased BBB permeability and ultimately vasogenic cerebral oedema; Secondly, activation of the hypoxia-inducible factor 1α/nuclear factor kappa B (HIF-1α/NF-κB) axis promotes aberrant release of inflammatory cytokines such as interleukin-1β (IL-1β) and tumour necrosis factor-α (TNF-α), instigating a neuroinflammatory cascade (Refs 13, 16). Targeting the metabolic disturbances induced by hypoxia will further significantly mitigate BBB disruption and cerebral oedema, thereby ameliorating HACE-induced cognitive dysfunction (Ref. 15).

### HACE and oxidative stress

Dysregulated of redox homeostasis permeates the entire pathogenesis of HACE, a pathological process that essentially represents an extension of damage stemming from energy metabolism disorders (Figure 1). Recent studies demonstrate that acute hypoxic exposure rapidly induces an abnormal elevation in ROS levels within brain tissue, with superoxide anion and hydrogen peroxide reaching peak levels as early as 2 hours post-exposure (Refs 15, 17). Concurrently, concentrations of lipid peroxidation products such as malondialdehyde and 4-hydroxynonenal show significantly increase, the levels of which are positively correlated with the degree of BBB disruption (Ref. 17). Notably, the antioxidant defence system undergoes phase-specific alterations during this process: initially, glutathione and superoxide dismutase levels decline sharply due to massive consumption, but a compensatory rebound occurs during the later stages of sustained hypoxia (Ref. 15). Furthermore, the underlying molecular regulatory mechanisms involve several key signalling pathways: (1) the HIF-1α and HIF-1β subunit-formed heterodimer initiates the expression of pro-oxidant genes by binding to hypoxia response elements (Ref. 18); (2) inhibition of JAK2/STAT3 or activation of the transcription factor FOXOs alleviates oxidative stress in HACE rat models (Refs 19, 20) and (3) activation of the transcription factor nuclear factor erythroid 2-related factor 2 (NRF2) and the PI3K/AKT-NRF2 pathway enhances systemic antioxidant capacity (Refs 21, 22). Notably, a significant reciprocal relationship exists between oxidative stress and inflammatory responses in HACE: ROS activate the IKKβ/NF-κB pathway, leading to a marked elevation in TNF-α and IL-6 levels, conversely, the inflammatory microenvironment exacerbates mitochondrial oxidative damage (Refs 17, 20).

### HACE and BBB injury

The development of cerebral cerebral oedema exhibits distinct stage-dependent evolutionary characteristics, with pathological progression divisible into two key phases: cytotoxic oedema and vasogenic oedema. Early-stage oedema is primarily triggered by osmotic imbalance driven by sodium ion (Na^+^) influx; the abnormal accumulation of Na^+^ induces passive transmembrane transport of chloride ions (Cl^−^) and water, consequently leading to swelling of neurons and glial cells. As the disease progresses, disruption of BBB integrity emerges as the predominant pathological feature, characterised by downregulated expression of endothelial tight junction proteins (e.g., ZO-1, Claudin-5) and abnormalities in the basement membrane structure. This ultimately results in plasma protein extravasation and the formation of vasogenic oedema, glia and neuron are damaged, which further exacerbates cerebral oedema and may be accompanied by haemorrhagic transformation in severe cases (Ref. 23). The aquaporin (AQP) family plays a pivotal role in the pathophysiological regulation of cerebral oedema. Among them, AQP4, the principal water transport molecule in the central nervous system, is specifically localised to astrocytic end-feet. Given that astrocytic endfeet constitute a critical component of the BBB, this localisation suggests an essential regulatory function for AQP4 in water homeostasis at the BBB level (Figure 1). Notably, AQP1 participates in intracranial pressure compensation by regulating cerebrospinal fluid secretion in choroid plexus epithelial cells. However, its mechanism of action is distinct from the AQP4-mediated water transport process across the BBB (Ref. 24). Experimental evidence demonstrates significantly elevated AQP4 expression in brain tissue of HACE models compared to control, concurrent with decreased Na^+^/K^+^-ATPase activity and degradation of tight junction proteins. This finding further corroborates the correlation between AQP4 overexpression and the severity of cerebral oedema (Refs 25, 26, 27). Furthermore, inhibition of AQP4 not only reduces transmembrane water flux but also mitigates BBB disruption by downregulating matrix metalloproteinase-9 (MMP-9) expression (Refs 25, 26, 28). Additionally, related mechanistic studies indicate that hypoxia excessively activates microglia, leading to disruption of endothelial tight junctions and swelling of astrocytes, thereby compromising BBB integrity and ultimately contributing to the induction of HACE (Refs 29, 30, 31).

### HACE and neuroinflammation

Neuroinflammation represents one of the primary etiological factors in HACE, with its core manifestations comprising hypoxia-triggered microglial activation and an inflammatory cascade (Figure 1). Research indicates that a hypoxic environment significantly activates microglia, driving the release of inflammatory mediators, including IL-1β, TNF-α and the chemokine CXCL1, via the TLRs/NF-κB signalling pathway, resulting in markedly increased expression levels of these factors (Refs 20, 32, 33, 34, 35). Concurrently, peripheral immune cells (such as monocytes and neutrophils) infiltrate the brain parenchyma through the compromised BBB, and mast cells aggregate in the hippocampal region, further exacerbating the local inflammatory microenvironment (Refs 34, 36). Studies have demonstrated that hypoxia-induced microglial activation leads to disruption of endothelial tight junctions and swelling of astrocytes. Furthermore, upregulation of nuclear respiratory factor 1 (NRF1) expression accelerates pro-inflammatory cytokine production by transcriptionally regulating NF-κB p65 and mitochondrial transcription factor A within hypoxia-activated microglia (Ref. 29). Notably, hypoxia-induced metabolic reprogramming substantially amplifies inflammatory effects; lactate accumulation promotes the transcription of inflammatory genes by inducing protein lactylation modifications (e.g., HDAC1) (Ref. 11). Additionally, elevated systemic inflammation combined with transient severe hypoxia form a synergistic regulatory axis involving AQP4 and TLR4/CRH receptor signalling, contributing to cerebral oedema development. CRHR1 antagonists may hold potential for the prevention and treatment of AMS and HACE (Ref. 37). Clinically, nonsteroidal anti-inflammatory drugs, such as ibuprofen, can be utilised to alleviate and prevent headaches during the onset of AMS (Refs 38, 39).

## Lactylation

### The discovery of lactylation

Lactylation is an emerging post-translational modification that plays a crucial role in regulating protein function and cellular metabolic networks. This mechanism involves the covalent attachment of a lactyl group to specific residues on target proteins. In 2019, Zhang et al. (Ref. 7) first identified histone lysine lactylation in a study published in *Nature.* They demonstrated that this modification directly enhances gene transcription by remodelling chromatin architecture. This groundbreaking discovery redefined lactate from merely a metabolic byproduct to an important epigenetic regulator. Subsequent research has shown that lactylation affects a wide range of substrates. For example, histone lactylation, such as at the H3K18 position, reprograms gene transcription through epigenetic remodelling. Additionally, non-histone lactylation influences gene expression, signal transduction and metabolic homeostasis by altering protein conformation, subcellular localisation and charge distribution. These findings offer new therapeutic targets and conceptual frameworks for disease intervention.

### The functions and regulation of lactylation

#### Functions of lactylation in disease progression

Lactylation modifications play crucial role in disease progression by regulating the tumour microenvironment (TME), influencing immune cell function, reprogramming metabolism, and facilitating signal transduction. Within the TME, lactylation contributes to tumorigenesis and metastasis by reshaping immune cell activity and promoting tumour spread. For example, the accumulation of lactate in the microenvironment induces histone H3K18 lactylation in tumour-infiltrating myeloid cells, which upregulates the expression of methyltransferase-like 3 and aids in tumour immune evasion (Ref. 40). Furthermore, lactylation at Lys72 of Moesin enhances the differentiation of regulatory T cells, suppresses antitumor immunity, and accelerates tumour growth (Ref. 41). Lactylation also influences macrophage polarisation to mitigate inflammation. Histone lactylation (e.g., H3K18la) promotes the expression of M2-phenotype-related genes (e.g., Arg1, IL-10) while downregulating M1 markers (e.g., iNOS, TNF-α) (Refs 7, 42). Non-histone lactylation is involved in this process as well; lactate-modified pyruvate kinase M2 (PKM2) shifts from dimeric to tetrameric forms, inhibiting glycolysis and alleviating impairments in oxidative phosphorylation, thereby driving M2 macrophage polarisation (Ref. 43). Additionally, lactylation influences disease progression by regulating the activity of metabolic enzymes and signalling pathways: The glycolytic reprogramming mediated by 6-phosphofructokinase/fructose-2,6-bisphosphatase 3 in renal tubular cells can enhance histone H4K12 lactylation, activating the NF-κB pathway and promoting renal fibrosis (Ref. 44). In hypoxia-induced colorectal cancer cells, β-catenin lactylation activates the Wnt/β-catenin signalling pathway, whereas downregulation of β-catenin significantly inhibits tumour proliferation (Ref. 45). In summary, lactylation modification serves as a metabolic-epigenetic regulatory hub involved in disease progression through mechanisms such as immune suppression, metabolic adaptation and oncogenic signalling.

#### Regulatory mechanisms of lactylation modification

The regulatory mechanism of lactylation is jointly determined by the dynamic balance of lactate metabolism and enzyme-dependent modifications, which collectively impart functional diversity. As a direct substrate, the intracellular concentration of lactate and its metabolic dynamics are among the factors driving lactylation modifications. Metabolic reprogramming, such as enhanced glycolysis or suppressed mitochondrial respiration, regulates the modification processes by altering lactate levels. Research has confirmed that the overall lactylation levels of both histone and nonhistone proteins are significantly glucose dose dependent (Ref. 46). Key enzymes involved in lactate metabolism also play a regulatory role in lactylation modification. For instance, sodium dichloroacetate can lower intracellular lactylation levels by activating pyruvate dehydrogenase (PDH), while LDH inhibitors can decrease LDH activity (Ref. 7). The regulatory effects of LDH on the lactylation process have been further validated in subsequent studies (Ref. 47). Notably, the threshold concentration of lactate that triggers lactylation modifications under different cell types or pathological conditions remains undefined; this heterogeneity may be related to tissue microenvironments, cellular metabolic characteristics, and experimental system differences. Additionally, exogenous lactate can enter cells through monocarboxylate transporters (MCTs), promoting pan-lactylation modifications (Refs 48, 49). The enzymatic system precisely regulates lactylation modifications through a dynamic balance between writer and eraser enzymes. The p300/CBP family of histone acetyltransferases can transfer lactyl groups to lysine residues on histones (such as H3K18la) or non-histones (such as YY1) using lactoyl-CoA under specific conditions (Refs 50, 51). Notably, p300-mediated H3K18 lactylation plays a crucial role in macrophage M2 polarisation (Ref. 7). Recent studies have expanded the types of writer enzymes, including HBO1, DPF2, and alanine-tRNA synthetase AARS1/2, which can act as intracellular L-lactate sensors to catalyse ATP-dependent lysine lactylation (Refs 52, 53, 54, 55). The eraser enzyme system includes members of the Sirtuin family (SIRT1–3, SIRT6) and histone deacetylase 2 (HDAC2), which reverse lactylation modifications on histones (such as H3K9la) and non-histones (such as β-catenin) through hydrolysis (Refs 56, 57, 58, 59, 60). In summary, the level of lactylation modification is determined by both the lactate metabolic flux and the activity ratio of writer to eraser enzymes. Lactate generation (e.g., glycolysis), transport (mediated by MCTs) and metabolism (regulated by LDH/PDH) directly influence substrate availability, whereas the enzymatic system dynamically regulates the functional states of target proteins through covalent modifications.

### Lactylation in different cell types

During hypoxic metabolism, pyruvate flux shifts from mitochondrial oxidative phosphorylation to cytosolic lactate production. This metabolic switch is driven by HIF-1α-dependent upregulation of glycolytic genes, including GLUT1 and LDHA, together with PDK1-mediated inhibition of the PDH complex (Ref. 18). The resulting lactate accumulation drives protein lactylation in various cell types. However, under hypoxic conditions, the mechanisms governing lactylation vary considerably among different brain cell types.

#### Microglia

As resident immune cells of the central nervous system, microglia employ lactylation to bridge metabolic state and immune function. While resting microglia rely primarily on oxidative phosphorylation, activation markedly upregulates aerobic glycolysis, resulting in intracellular lactate accumulation and widespread protein lactylation (Ref. 61). During acute disease phases, lactate-induced lactylation predominantly promotes microglial activation and inflammatory responses. For instance, H3K18 lactylation enhances Rela promoter activity and NF-κB1 binding, thereby activating the NF-κB pathway and amplifying inflammation (Ref. 62). PKM2 and LDHA, key rate-limiting enzymes in glycolysis and lactate production, are critical in this process. For example, in Alzheimer’s disease (AD) mice, H4K12 lactylation accumulates at promoters of glycolytic genes in microglia, increasing glycolytic activity and microglial activation (Ref. 63). Notably, identical modification sites can exert opposing functions depending on the pathological context. In cigarette smoke-induced AD mice, H4K12la accumulation at the NLRP3 promoter activates the inflammasome, subsequently impairing microglial autophagy (Ref. 64). Conversely, in a spinal cord injury model, lactate-mediated H4K12la elevation promotes PD-1 transcription in microglia, facilitating injury repair (Ref. 65). Furthermore, altered microglial lactylation levels influence astrocyte precursor cell generation during mouse brain development (Ref. 66). These findings indicate that the functional diversity of microglial lactylation stems from disease- and stage-specific contexts, acting as a multifaceted regulator in the dynamic balance between neuroinflammation and tissue repair.

#### Astrocytes

Astrocytes, which construct the BBB, maintain ion homeostasis, and regulate synaptic transmission, also depend on lactylation as a core regulatory factor in injury responses. Similar to microglia, astrocyte lactylation exhibits a ‘double-edged sword’ characteristic in immune polarisation. On one hand, lactylation can drive conversion to an inflammatory phenotype; for example, H3K18 lactylation regulates LCN2 m6A modification following intracerebral haemorrhage in mice, promoting A1 astrocyte polarisation (Ref. 67). Conversely, in oxygen–glucose deprivation/reoxygenation (OGD/R) models, astrocytic lactylation regulates migration and A2 polarisation (Ref. 68). This suggests that lactylation acts primarily as an environment-sensitive ‘accelerator’ in astrocyte immune responses, with its ultimate effect determined by the nature of upstream signalling inputs. As the primary source of brain lactate, astrocytic lactylation profoundly influences intercellular interactions and neuronal survival. Astrocytes can promote mitochondrial transfer to neurons and alleviate neuronal injury by reducing lactate production and ARF1 lactylation (Ref. 69). However, increased astrocyte-derived brain lactate can exacerbate ischaemic brain injury by promoting protein lactylation in brain tissue; mice with astrocyte-specific LDHA knockout show significantly downregulated lactylation and reduced infarct volume compared to controls (Ref. 70). Meanwhile, H4K8 lactylation in astrocytes activates glycolysis-related genes, further increasing lactate production to ensure neuronal energy supply and promote motor function recovery after spinal cord injury (Ref. 71). Thus, astrocytes are not merely lactate producers; their own lactylation status is a key variable determining neural microenvironment homeostasis and injury outcomes.

#### Neurons

In neurons, lactylation primarily regulates neurite outgrowth, stem cell differentiation, and cell death fate. Regarding structural plasticity, α-tubulin lactylation directly promotes axonal growth and dendritic branching in hippocampal neurons, providing a structural basis for neural network reconstruction (Ref. 72). At the developmental and regenerative levels, exogenous lactate supplementation under hypoxic conditions drives neural stem cell differentiation into the neuronal lineage by inducing H3K9 lactylation, and this process is completely abolished when this modification is inhibited (Ref. 73), highlighting the necessity of lactylation in neurogenesis. Simultaneously, lactylation serves as a critical molecular switch determining neuronal survival or death, with effects highly dependent on target protein specificity. In traumatic brain injury models, H3K18la enrichment at the PSMD14 promoter upregulates PSMD14 expression, which effectively inhibits neuronal apoptosis by enhancing mitochondrial capacity to clear ROS (Ref. 74). In contrast, in haemorrhagic stroke, H3K14la induces ferroptosis by transcriptionally activating the ATP2B2 gene, leading to intracellular calcium overload (Ref. 75).

## Lactate, lactylation and HACE

### Glycolysis and lactate

Under normal physiological conditions, glucose serves as the primary energy source for the human brain. It is metabolised to pyruvate within both astrocytes and neurons. Subsequently, pyruvate undergoes predominantly mitochondrial aerobic oxidation, yielding substantial energy to support neuronal activity. However, under pathological conditions, anaerobic glycolysis in the cytosol becomes the primary source of cellular ATP in such contexts (Ref. 76).

Traditionally, lactate was considered merely a metabolic waste. However, recent studies have fundamentally revised this misconception regarding lactate’s physiological significance. Firstly, lactate serves as an energetic substrate through the astrocyte-neuron lactate shuttle (ANLS) mechanism. Under conditions of intense exercise or hypoxia, anaerobic metabolism escalates, leading to a 180.3% increase in cerebral lactate concentration (Ref. 77). This lactate, released from glial cells, is subsequently taken up by neurons where it serves as a preferential oxidative substrate over glucose for ATP production, potentially accounting for 33% of total cerebral energy substrates (Refs 77, 78, 79). Secondly, lactate functions as a signalling molecule by activating the G protein-coupled receptor 81 (GPR81), thereby modulating neuronal activity and energy metabolism (Ref. 80). Studies demonstrate that GPR81 activation by lactate participates in developmental cerebrovascular angiogenesis and mitigates neonatal hypoxic–ischaemic (HI) injury by restoring compromised microvasculature and enhancing neurogenesis. These multifaceted roles of lactate likely play pivotal roles in the oxygen-sensitive brain, particularly in hypoxia-related pathologies (Refs 81, 82).

### The role of lactate in HACE

In an acute hypoxic environment, the body’s lactate metabolism exhibits significant dynamic changes. A prospective trial demonstrated that upon arrival at a high altitude of 4559 m, plasma lactate levels increased in a time-dependent manner. Specifically, compared to baseline values at low altitude, mean fasting blood lactate levels showed an increasing trend after 2 days of exposure (MG2). Following 4 days of sustained exposure (MG4), however, both fasting and postprandial lactate responses exhibited a statistically significant increase. These findings indicate that with prolonged hypoxic exposure, metabolism is progressively activated, with catabolism becoming predominant (Ref. 83). Similarly, when five subjects were exposed to a hypoxic chamber simulating an altitude of 6706 m, the lactate concentration in the subcortical white matter of the brain increased (Ref. 84). Notably, subjects in the inhaling hypoxic air group showed increased lactate and cerebral blood flow, with synchrony between the increase in brain tissue lactate concentration and cerebral blood flow, while their cerebral metabolic rate remained stable, suggesting that lactate may improve oxygen supply by activating GPR81-mediated cerebrovascular angiogenesis (Refs 85, 86). Animal experiments further confirmed that hypobaric hypoxia (simulating an altitude of 6000 m) or combined with lipopolysaccharide stimulation can significantly increase the lactate concentration in the hippocampus, and it is positively correlated with the increase in BBB permeability (Refs 11, 15).

The accumulation of lactate is closely associated with hypoxic adaptive regulation. Under hypoxic conditions, oxidative phosphorylation is suppressed while glycolytic ATP production yields less than 1/10 of complete glucose oxidation. The compensatory strategy involves further metabolic suppression and enhanced glycolysis, so elevated lactate is related to individual brain tolerance under hypoxia. Mild acidosis modulates mitochondrial dynamics and cristae structure to remodel mitochondria and reconfigure their efficiency (Ref. 87). in vitro studies demonstrate that cortical neurons in weakly acidic environments (pH ≤ 6.8) exhibit greater viability with intact mitochondria compared to those in hypoxic neutral conditions (pH 7.2–6.8), suggesting this may represent an endogenous survival strategy during acute hypoxia (Refs 87, 88). Moreover, the MCTs-dependent lactate shuttle plays an important role in high-altitude adaptation, where upregulated MCT1/4 gene expression reduces central fatigue (Refs 89, 90). Both acetazolamide and *Rhodiola rosea* are commonly used for preventing and treating high-altitude illnesses, and pathway enrichments associated with glycolysis/gluconeogenesis (Ref. 91). Administration of salidroside under normoxic conditions can also activate compensatory responses by activating HIF-1α signalling pathway, elevating lactate levels, enhancing glycolytic capacity while attenuating oxidative phosphorylation, ultimately improving neurological function (Ref. 92).

Lactate plays a complex role in hypoxic encephalopathy. A neonatal rat HI model showed that exogenous lactate intervention significantly reduced the volume of brain injury and improved sensorimotor and cognitive behaviours (Refs 93, 94). However, lactate receptor HCAR1 knockout mice exhibited cell cycle disorders and immune-inflammatory responses, and after HI injury, brain tissue of HCAR1-deficient mice produced almost no new cells, suggesting that lactate signalling regulates tissue repair (Refs 81, 82). Similarly, the LDH inhibitor oxalate completely reversed the neuroprotective effect of lactate, confirming that its action depends on the integrity of the lactate metabolic pathway (Ref. 95). in vitro models of stroke have shown that exogenous administration of lactate before hypoxia has a neuroprotective effect and is associated with the ANLS (Ref. 96). Additionally, lactate administration may serve as a potential adjuvant therapy for gliomas, holding therapeutic applications. Studies have found that lactate-loaded nanoparticles induce glioma cell cytotoxicity and improve the survival rate of rats with malignant glioma brain tumours (Ref. 97). Conversely, the presence of lactate may also be associated with poor outcomes. An investigation of 47 brain lesions revealed that increased lactate concentrations in stereotactic puncture samples may indicate poor prognosis and increased astrocytoma grade (Ref. 98). Clinically, serum lactate may be a useful biomarker for improving early ICU treatment in patients with isolated traumatic brain injury. Patients with elevated lactate levels on admission require higher cardiopulmonary support within the first 24 h in the ICU to reduce tissue hypoperfusion and alleviate hypoxic status (Ref. 99). The key glycolytic enzyme hexokinase 2 induces neuroinflammation and neurotoxicity under hypoxia in stroke models (Ref. 61).

Collectively, these related studies have revealed the roles of glycolysis and lactate in hypoxic states, including regulating diseases as signalling molecules, the ANLS-based energy metabolism transport mechanism, and metabolic reprogramming. However, the specific effects may exert different roles depending on the degree of hypoxia, pathological processes and microenvironment, and largely reflect their importance in the treatment or prevention of HACE, which warrants further investigation, particularly regarding the new research avenue of lactate**–**lactylation modification.

### Lactylation – A new target for HACE

**Figure 2.**
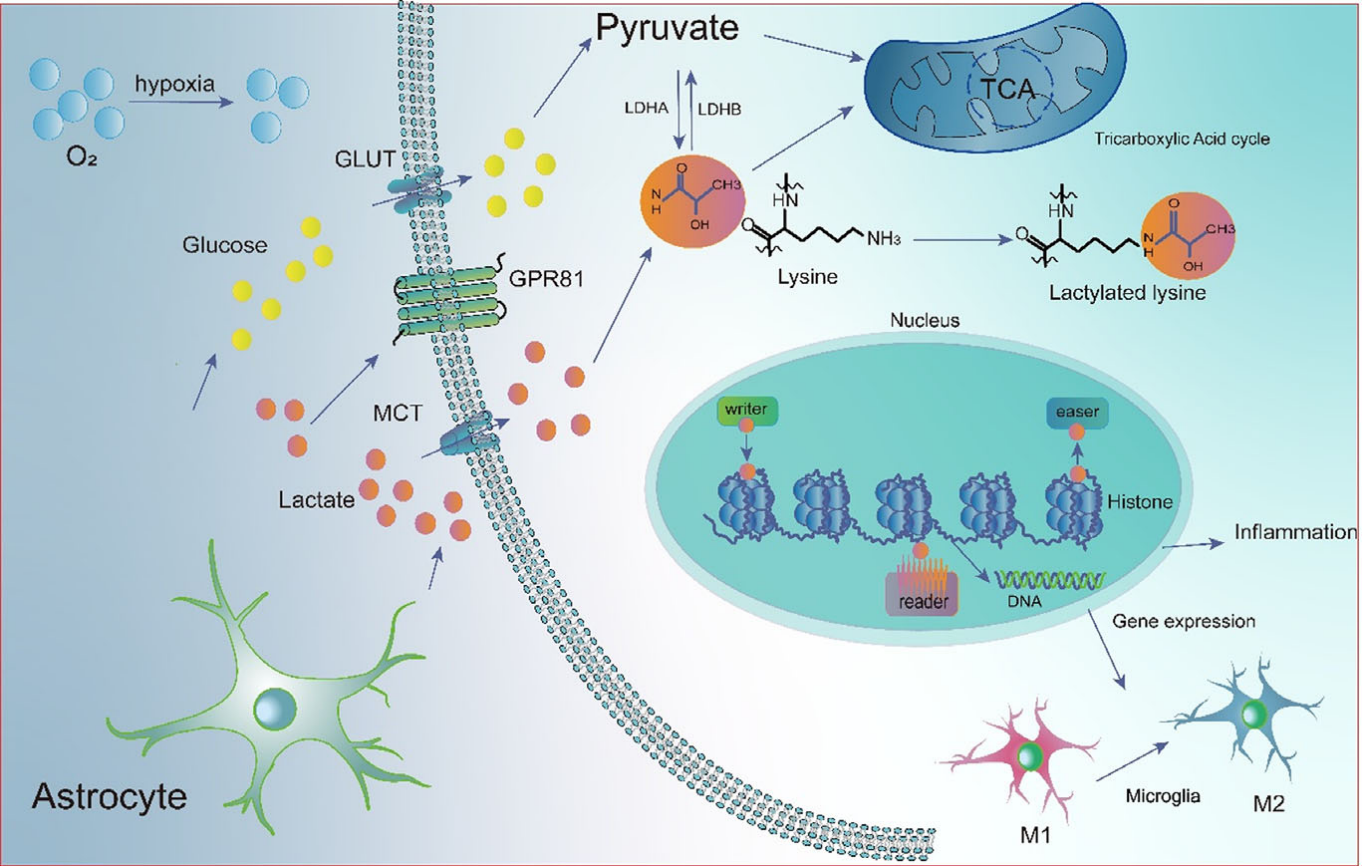
Schematic diagram of cerebral lactate metabolism and lactylation mechanisms under high-altitude hypoxia. Under normoxic conditions, glucose enters cells via GLUTs and is metabolised to pyruvate, which enters mitochondria for the TCA cycle and ATP production. Under hypoxia, pyruvate is converted to lactate by LDHA. Lactate from circulation or astrocytes enters cells via MCTs and is used for histone lactylation: it is converted to lactyl-CoA and transferred to lysine residues by writer enzymes (e.g., p300). Reader proteins recognise these marks to regulate gene expression, while eraser enzymes (e.g., HDACs) remove them. Notably, this lactylation ‘clock’ regulates immune homeostasis by influencing Microglia polarisation, potentially driving the transition from pro-inflammatory M1 to anti-inflammatory M2 phenotypes. (Abbreviations: TCA: tricarboxylic acid; LDHA: lactate dehydrogenase A; MCTs: monocarboxylate transporters; GLUTs: glucose transporters).

Lactate, a key metabolic byproduct of hypoxia, plays a significant regulatory role in the progression of HACE. Clinical studies have confirmed that plasma lactate concentrations significantly increase in healthy subjects after 8 hours of simulated altitude exposure at 5500 m (Ref. 100). Metabolomic analysis of blood from individuals ascending rapidly to 4,300 m similarly demonstrated significant increases in both D-lactate and L-lactate levels (Ref. 101). Furthermore, patients with AMS exhibit higher blood lactate levels compared to healthy controls (Ref. 102). Leandr and colleagues reported a positive correlation between blood lactate values and AMS scores; specifically, subjects with higher blood lactate levels at 5,000 m had a 23% increased risk of developing moderate AMS compared to those with lower levels (Ref. 103). This lactate accumulation stems not only from altered metabolic substrates but also from the adaptive upregulation of key enzymes. Proteomic analyses reveal significantly enhanced expression of LDHA, the rate-limiting enzyme of glycolysis, in both AMS patients and HACE patients at 4,500 m. Notably, LDH levels in HACE patients upon admission exceed those in patients with HAPE (Refs 104, 105). These findings suggest that lactate is not merely a byproduct of hypoxic metabolism but a critical signalling molecule driving HACE pathology (Figure 2). Recent studies further elucidate that lactate directly regulates gene expression via a novel post-translational modification known as lactylation, serving as a core mechanism for the body’s response to hypoxic stress. Lactylation profoundly influences HACE development through a ‘metabolism-inflammation’ positive feedback loop. For instance, under acute hypoxic conditions, excessive mitochondrial ROS production activates the HIF-1α/PDK1&PDK2/p-PDH-E1α pathway, which promotes histone lactylation and drives proliferation and vascular remodelling in pulmonary artery smooth muscle cells (Ref. 106). ChIP-seq analysis confirmed that H3K18la is significantly enriched in the promoter regions of HIF-1α target genes (e.g., Bmp5, Trpc5, Kit), facilitating the proliferation and vascular remodelling of pulmonary artery smooth muscle cells (Ref. 106). Additionally, H3K18 lactylation directly activates the NF-κB signalling pathway (Ref. 62). This mechanism likely applies to cerebrovascular lesions in HACE, where H3K18la may exacerbate cerebral oedema by opening chromatin structures of pro-inflammatory genes or genes related to vascular permeability. Lingling Zhu and colleagues, through in-depth proteomic analysis of microglia in a HACE mouse model, revealed extensive lactylation of the NuRD chromatin remodelling complex, a key regulator of gene transcription and inflammation. They identified 62 lactylation sites within its core subunits, including HDAC1, MTA1, and GATA2B. Further assays indicated that lactylation at these sites alters the proteins’ 3D structure and inhibits HDAC1 enzymatic activity, potentially derepressing the transcription of inflammatory genes such as TNF-α and IL-1β. Consequently, abnormal lactylation of the NuRD complex may represent a key molecular mechanism exacerbating microglial neuroinflammatory responses under hypoxic conditions, thereby promoting HACE progression (Ref. 11). The levels of these modifications correlated positively with pro-inflammatory cytokine expression, suggesting that lactylation modification may play a key role in the pathogenesis of HACE. Notably, under hypoxic conditions, lactylation of HIF-1α blocks VHL recognition, enhancing its stability (Ref. 107). HIF-1α can subsequently activate NF-κB and p53 pathways, elevating neuroinflammation and promoting cerebral oedema (Refs 27, 108). It has been confirmed that novel saligenin derivatives, acting as potent HIF-1α signalling inhibitors, can treat HACE in mice (Ref. 109). Given that VEGF, a downstream molecule of HIF-1α, plays a critical role in BBB disruption in HACE, inhibiting HIF-1α lactylation may reduce BBB injury by lowering VEGF production (Ref. 110). Lactylation shows marked kinetic delay, peaking after lactate accumulation. In mice after a single bout of intermittent high-intensity exercise, lactate rose rapidly but tissue lactylation peaked only at 24 hours post-exercise, then gradually decreased (Ref. 111). This delay was also observed in HACE models: both cortical tissues from HACE mice and BV2 cells showed lactylation peaking at 24 hours after hypoxic exposure (Ref. 112). This 24-hour peak coincides with the clinically observed onset window for HACE (Ref. 113). Based on these observations, we infer that during initial high-altitude exposure, brain tissue undergoes metabolic adaptation through enhanced glycolysis, leading to sustained lactate generation and accumulation. This persistent metabolic stress drives progressive lactylation elevation, reaching a critical threshold at 24 hours. At this point, high lactylation levels act as an epigenetic switch, triggering robust activation of downstream pro-inflammatory gene transcription, inducing an inflammatory storm and disrupting the BBB, ultimately leading to HACE.

Given the critical role of lactylation in HACE, targeting this pathway offers significant therapeutic potential, though its bidirectional regulatory nature must be considered. In terms of acute intervention, the primary strategies involve reducing endogenous lactate levels or directly blocking lactylation. Reducing endogenous lactate levels (e.g., through the use of LDH inhibitors or MCT inhibitors) may effectively alleviate acidosis and inflammatory responses dependent on lactylation modification (Refs 114, 115). However, caution is advised, as non-specific inhibition could disrupt normal lactate metabolism. Additionally, classical deacetylases (HDAC1–3 and SIRT1–3) have delactylase activity and may provide therapeutic benefits by restoring protein functional balance (Ref. 116). The small-molecule blocker D34–919, which inhibits the glycolysis-lactylation signalling pathway by disrupting the interaction between ALDH1A3 and PKM2, has shown effectiveness in glioma models, highlighting its potential applicability in HACE treatment (Ref. 117). Utilising nanocarriers (such as SS-HPT) to deliver HIF-1α-shRNA can significantly reduce lactylation levels under sustained hypoxia and ameliorate pulmonary dysfunction (Ref. 118), this strategy could be adapted for HACE treatment. The commonly used clinical drug dexamethasone has also been found to downregulate the HIF-1α glycolytic axis and inhibit lactylation in asthma models (Ref. 119), suggesting that its new mechanism for treating HACE may partly stem from suppressing the lactylation pathway. Notably, while excessive lactylation during the acute phase acts as a pathological pro-inflammatory driver, moderate physiological lactate elevation during prevention and recovery phases may exert neuroprotective effects. Research indicates that exogenous lactate supplementation significantly reduces neurological impairment in neonatal HI models (Ref. 93). Furthermore, in Alzheimer’s disease and aging models, exercise-induced lactate elevation enhances histone H3K18 lactylation, promoting the shift of microglia from a pro-inflammatory M1 phenotype to an anti-inflammatory M2 phenotype, thereby reducing neuroinflammation (Ref. 120). In mice recovering from spinal cord injury, exercise performed before and during the recovery phase elevated lactate levels, exerting protective effects via lactylation (Ref. 121). These findings suggest that lactylation plays a ‘double-edged sword’ role in HACE: pathological lactylation during the acute phase warrants inhibition, whereas physiological lactylation during prevention or recovery should be preserved or promoted. Therefore, future therapeutic strategies should not be limited to simple ‘inhibition’ but should shift towards precise regulation. This involves developing specific inhibitors to block the vicious inflammatory cycle in the acute phase, while simultaneously exploring methods such as scientific exercise or exogenous lactate pretreatment to induce protective lactylation, providing new intervention strategies for the prevention and prognosis of HACE.

## Conclusion

As the terminal stage of acute mountain sickness, the pathological mechanisms of HACE involve multiple aspects induced by acute hypoxia, including energy metabolism disorder, oxidative stress, neuroinflammation and BBB disruption. In recent years, lactate metabolism and its derived lactylation modification have gradually emerged as key entry points for understanding the pathogenesis of hypoxic diseases. This paper systematically reviews the dual roles of lactate in hypoxic environments. More importantly, as a novel epigenetic regulatory mechanism, lactylation modification directly participates in gene transcription regulation, inflammatory responses and metabolic reprogramming under hypoxic stress by dynamically modifying histone and non-histone targets.

The importance of lactylation in HACE has become increasingly apparent; however, research has focused primarily on the general role of lactylation during HACE progression, with limited investigation into the mechanisms of specific modification sites. Therefore, we have compiled lactylation sites and their target cell types in hypoxic brain diseases for reference (Table 1). Additionally, existing studies still have limitations: (1) The dynamics of lactylation during HACE onset, progression, and recovery remain poorly defined, with a lack of spatiotemporal omics analyses to identify critical disease and therapeutic windows. (2) Clinical translation is hampered by a paucity of direct evidence from HACE patient cerebrospinal fluid, blood or post-mortem tissues; most conclusions rely on animal models or in vitro studies. (3) Cell-specific responses and molecular mechanisms remain unclear. Distinct brain cell types (microglia, astrocytes, neurons and endothelial cells) exhibit heterogeneous responses to lactylation. Moreover, critical lactate concentration thresholds, specific modification sites and the enzymatic machinery responsible for writing and erasing these marks (writers such as p300 and GCN5, erasers such as HDACs) remain to be elucidated in HACE. (4) Current intervention strategies lack specificity. Potential therapies such as LDH inhibitors or MCT blockers globally suppress lactate metabolism, risking disruption of essential physiological processes including the ANLS and causing severe metabolic side effects.

**Table 1.** The potential targets of lactylation modification in HACE

Cell/Tissue/Species	Modified Gene/Site	Related disease	Mechanism	Ref.
Rat brain microvascular endothelial cells (RBMVECs)	H3K18la	IS	Reduced H3K18la, which suppresses Apaf–1 transcription, attenuates apoptosis and protects the neurovascular unit	(Ref. 122)
Mouse BV2 cells; C57BL/6 mice	H3K18la	IS	H3K18la promotes plxnb2 expression, regulates microglial phenotype, alleviates neuroinflammation	(Ref. 123)
Mouse primary astrocytes, neurons; C57BL/6 mice	tau K56	IS	Acetate promotes astrocytic glucose uptake and glycolysis, stimulates ANLS, promotes neuronal tau protein K56 lactylation, enhances neuronal plasticity	(Ref. 124)
Mouse BV2 cells; SD rats	PLBD1 K155	IS	Ischaemia-induced lactate accumulation promotes PLBD1 K155 lactylation, enhances its stability, promotes glycolysis and neuroinflammation	(Ref. 125)
C2C12 cells/HL–1 cells; C57BL/6 mice	PDHA1 K336; CPT2 K457/8	Hypoxia, Exercise	Hypoxia induces AARS2 expression, which acts as a lactyltransferase, lactylating PDHA1 K336 and CPT2 K457/8, inhibiting their activity and limiting OXPHOS	(Ref. 126)
Mouse BV2 cells	H3K9la	MCAO/R	SMEK1 deficiency inhibits the PDK3/PDH pathway, increases H3K9la, activates Ldha and Hif–1α transcription, promotes glycolysis and lactate production	(Ref. 127)
Rat PASMCs; SD rats	H3K18la; H4K5la	PH	mROS-mediated HIF–1α stabilisation upregulates PDK1/2, promotes lactate accumulation, H3K18la and H4K5la, activates Bmp5, Kit, Trpc5, etc.	(Ref. 106)
Mouse primary astrocytes, neurons; C57BL/6 J mice	ARF1K73	IS	LRP1 inhibits glycolysis and lactate production, reduces ARF1K73 lactylation, promotes mitochondrial release to neurons	(Ref. 69)
Human scleral fibroblasts; C57BL/6 mice; Guinea pigs	H3K18la	Myopia	Hypoxia induces glycolysis and lactate, promotes H3K18la, activates Notch1, induces FMT	(Ref. 128)
Mouse N2a cells; SD rats	H3K18la	CI/R	LDHA upregulation promotes lactate, increases H3K18la, activates HMGB1, induces pyroptosis	(Ref. 129)
Rat PC12 cells; SD rats	LCP1	IS	Ischaemia-induced lactate accumulation promotes LCP1 lactylation, exacerbates energy metabolism disorder and apoptosis	(Ref. 130)
Neuron cell; C57BL/6 mice	Crmp1	IS	Astrocyte-derived lactate drives neuronal protein lactylation, exacerbates injury	(Ref. 70)

Abbreviations: CI/R: Cerebral ischaemia reperfusion; IS: Ischaemic stroke; MCAO/R: Middle cerebral artery occlusion and reperfusion; PH: Pulmonary hypertension.

In summary, lactylation provides a novel perspective on HACE pathogenesis, and its apparent bidirectional regulatory nature suggests considerable potential for targeted therapy. Future research should prioritise: (1) Employing time-resolved proteomics combined with single-cell sequencing to systematically map lactylation dynamics across brain regions and cell types during HACE, identifying critical time points and cellular subpopulations. (2) Using CRISPR/Cas9 to generate mouse models with site-specific mutations, thereby validating in vivo the role of specific sites in regulating BBB permeability, neuroinflammation and oedema formation. (3) Developing targeted small-molecule drugs or nanoparticle delivery systems that specifically modulate lactylation, rather than indiscriminately blocking lactate production. (4) Determining whether exercise preconditioning, staged ascent training or exogenous lactate supplementation can induce protective lactylation and thereby prevent HACE.

## Data Availability

No datasets were generated or analysed during the current study.
